# Effects of Stocking and Transport Conditions on Physicochemical Properties of Meat and Acute-Phase Proteins in Cattle

**DOI:** 10.3390/foods10020252

**Published:** 2021-01-26

**Authors:** Ahmed A. Abubakar, Idrus Zulkifli, Yong M. Goh, Ubedullah Kaka, Azad B. Sabow, Jurhamid C. Imlan, Elmutaz A. Awad, Azalea H. Othman, Razlina Raghazli, Helen Mitin, Awis Q. Sazili

**Affiliations:** 1 Institute of Tropical Agriculture and Food Security, Universiti Putra Malaysia (UPM), Serdang 43400, Selangor, Malaysia; ahmadsadeeq7@gmail.com (A.A.A.); zulidrus@upm.edu.my (I.Z.); ymgoh@upm.edu.my (Y.M.G.); jurhamidimlan@yahoo.com.ph (J.C.I.); elmutazatta@upm.edu.my (E.A.A.); helenmitin21@gmail.com (H.M.); 2 Department of Animal Science, Faculty of Agriculture, Universiti Putra Malaysia (UPM), Serdang 43400, Selangor, Malaysia; 3 Department of Preclinical Sciences, Faculty of Veterinary Medicine, Universiti Putra Malaysia (UPM), Serdang 43400, Selangor, Malaysia; razlina81@gmail.com; 4 Department of Companion Animal Medicine and Surgery, Faculty of Veterinary Medicine, Universiti Putra Malaysia (UPM), Serdang 43400, Selangor, Malaysia; dr_ubedkaka@upm.edu.my; 5 Department of Animal Resource, College of Agriculture, Salahaddin University-Erbil, Kurdistan Region 44002, Iraq; azad.sabow@su.edu.krd; 6 Department of Animal Science, College of Agriculture, University of Southern Mindanao, Kabacan 9407, North Cotabato, Philippines; 7 Department of Veterinary Laboratory Diagnostics, Faculty of Veterinary Medicine, Universiti Putra Malaysia (UPM), Serdang 43400, Selangor, Malaysia; azalea@upm.edu.my; 8 Department of Veterinary Services, Wisma Tani, Blok Podium, Lot 4G1, No. 28, Persiaran Perdana, Presint 4, Federal Government Administrative Centre, Putrajaya 62630, Malaysia; 9 Halal Products Research Institute, Universiti Putra Malaysia (UPM), Serdang 43400, Selangor, Malaysia

**Keywords:** Brahman crossbred, hot humid climate, road transport, meat quality, Malaysia

## Abstract

This study’s objective was to evaluate the effects of distance and stocking density on physicochemical properties and oxidative stability of meat and acute-phase proteins in Brahman crossbred cattle transported by road under hot and humid tropical conditions. Sixty Brahman crossbred heifers were subjected to road transport from a cattle feedlot farm located in Universiti Putra Malaysia (UPM), Serdang, to a commercial ruminant abattoir in Shah Alam, Selangor. Animals were assigned to long and short distances and high, medium, and low stocking densities. The results revealed that the intensity of response significantly increased in meat samples from animals subjected to long-distance transportation and higher stocking density. Alpha-1-acid glycoprotein and serum amyloid-A values increased considerably and were different from the baseline values recorded at preload. In conclusion, the current results revealed that the color, pH, shear force values, water holding capacity (WHC), glycogen level, and malondilaldehyde assay (MDA) concentrations in meat and acute-phase proteins (APP) were affected by both distances and stocking densities, as evidenced by the significant changes recorded from the parameters above.

## 1. Introduction

The intensity and specialization of livestock production in particular areas and the demand for them to be marketed and slaughtered in other places where they are not reared have necessitated transportation of food animals all over the world [1,2]. Stress factors associated with transportation can be categorized into the “short-acting” factors that tend to have emotional effects on animals and the “long-acting” factors that have physical consequences and can accumulate over time [3].

Transportation is a critical point in the meat production system, whose time and mismanagement may pose risks to animal welfare and meat quality. Distance and duration of the journey are the aspects that can affect animal welfare during transport from the farm to the slaughterhouse [4,5,6]. Transportation does not only affect animal welfare, but it can also negatively influence meat quality and cause economic losses [6,7,8,9]. Thus, it is highly necessary to control and minimize transport-related stress-inducing factors for ethical, quality, and economic reasons [10].

Ayo and Minka [11] reported that a reduction in ATP before the slaughter of animals, due to variable durations of transport, led to the depletion of muscle glycogen, which inversely increased plasma glucose production. The depletion of muscle glycogen reserves preslaughter has a profound and well-documented effect on several key meat quality attributes such as tenderness and ageing potential, color, and water-holding capacity [12].

Previous research has studied the effects of transport-related factors such as transport duration, stocking density, and weather on the meat quality in lambs [13,14,15], pigs [16,17], and rabbits [18].

Transportation is one of the most widely recognized beef production stressors [19,20]. Numerous works have evaluated the influence of transport-related factors associated with the welfare of cattle [21,22,23,24]. Nevertheless, information on the effects of distance and stocking density during transport on meat quality attributes and acute-phase proteins in cattle, under tropical conditions, is scarce.

According to a report by the Malaysian Department of standards [25], self-sufficiency, in terms of beef production (SSL), is 27.5% and largely depends on imports from neighbouring countries such as Thailand Indonesia to meet up with its surging demand. In addition, these animals are transported mainly by road to various feedlots and slaughter plants, which is a call for concern as to how they are handled, transported, and slaughtered.

According to a report in 2017, Malaysia imported 25,585 live animals from Australia, of which 23,542 were beef cattle on a journey that necessarily involved both sea and long-distance road transport [26]. Additionally, the Animal Welfare Act 2015 came into existence after thorough legislation, which requires detailed scientific evidence to develop a comprehensive guideline for livestock handling, transportation, and management. It can only be achieved through the conduct of a trial. The current study results will add to the implementation of Acts and regulations for animal transportation and the Animal Welfare Act 2015 [27]

Thus, the present study was undertaken to investigate the effects of distance and stocking density on physicochemical properties and oxidative stability of meat and acute-phase proteins in Brahman crossbred cattle transported by road under the hot and humid tropical conditions.

## 2. Materials and Methods

### 2.1. Ethical Note

The experimental protocol was approved by the Institutional Animal Care and Use Committee (IACUC) of the Universiti Putra Malaysia (UPM/IACUC/R028/2016).

#### 2.1.1. Housing and Management

All animals were housed in naturally ventilated pens (15 animals per pen) with concrete flooring and PVC roofing. The floor space was 3.5 m^2^/animal. The animals had ad libitum access to commercial beef cattle feed, grass pellets, rice straw, and drinking water, and lighting for 24 h. The average in-house daily mean maximum and minimum daily dry bulb temperatures and relative humidity were 33.0 ± 1.36 °C during the day and 23.1 ± 1.40 °C at night, and relative humidity was 82.6 ± 1.40%, respectively (Malaysian Department of Meteorology) [28].

#### 2.1.2. Animals, Transport, and Treatment

Sixty Brahman crossbred heifers of about 24 months of age and a live weight of about 290.0 ± 36.0 kg were allotted into two transport distances of 450 km (medium distance) and 850 km (long distance). Three stocking densities with 10 animals each were assigned to either 600 kg/m^2^ (high SD), 400 kg/m^2^ (medium SD) and 200 kg/m^2^ (low SD). Animals were transported for either 9 h (medium distance) or 17 h (long distance) before been unloaded at a commercial ruminant abattoir for slaughter. The departure times from the farms and arrival times at the abattoir for the cattle were recorded. All animals were transported using the highway. Then, the transportation duration for randomly selected animals was calculated as a difference between arrival time and departure time. The mean daily temperatures at the farm and other parts of the state the animals crossed during the road transportation ranged between 32 and 35 °C during the day and 22 and 24 °C at night. The relative humidity was (84.1%); the data were obtained from the Malaysian Meteorological Department [28].

#### 2.1.3. Blood Sampling

All animals were sampled via the jugular vein with 18-G needles and a 10 mL vacutainer tube (BD Franklin Lakes, NJ, USA) with an aseptic precautionary measure. The blood samples were collected at preload, unload, and at slaughter (post neck cut from blood flow) and temporarily stored on ice. Serum samples were separated by centrifugation at 1600× *g*, for 15 min, at 8 °C and subsequently stored at −80 °C until subsequent analysis.

#### 2.1.4. Slaughtering Procedure

Slaughtering was carried out at a commercial ruminant abattoir, in Shah Alam, Selangor Darul Ehsan. Within 30 min upon arrival, animals were properly unloaded and rested in the lairage area for 12 hours, where an unlimited amount of drinking water was provided to them before slaughter. After the lairage, animals were transferred through a solid concrete race or conveyer and carefully restrained in a restraining box. All animals were subjected to Halal slaughter without stunning, as outlined in the MS1500: 2009 (Department of Standards Malaysia, 2009) [25]. The process involved severing the two carotid arteries, jugular veins, trachea, and oesophagus.

#### 2.1.5. Muscle Sampling and Storage

Within 30 min postmortem, the right *Longissimus lumborum* (LL) muscle was removed and divided into two parts. The first portion was placed in a Dewar flask and snap-frozen in liquid nitrogen (Malaysian Oxygen Pty. Ltd., Malaysia) before being stored at −80 °C until subsequent determinations pre-rigour muscle pH, glycogen content, and lipid oxidation. The samples were snap-frozen to prevent further biochemical changes, particularly in the pre-rigour muscle. Simultaneously, the second portion was vacuum packed and directly stored at −80 °C and assigned for subsequent determinations of cooking loss, shear force, and color at d 0 postmortem. The carcasses were hung in the cold room at 4 °C, until the next sampling was done at the following three (3) specific periods: 1, 7, or 14 days postmortem. Upon completing each ageing period, the samples were dissected from the LL muscle, vacuum packed, and transferred to a −80 °C freezer and stored until subsequent analyses of glycogen, pH, color, cooking loss, shear force, and lipid oxidation.

#### 2.1.6. Color

A ColorFlex spectrophotometer (Hunter Lab, Reston, Virginia, USA) based on the International Commission on Illumination (CIE) was used to determine meat color values with laboratory values (also known as L*, a*, b*), using a D56 illuminant and a 10° standard observer, tristimulus values (X, Y, Z) as reflectance at a specific wavelength of 400–700 nm to express the meat color data. Before use, the colorimeter was calibrated against black and white tiles. The frozen LL muscle samples for days 0, 1, 7, and 14 were transferred from −80 °C freezer into a 4 °C chiller overnight. The thawed samples of approximately 12 mm of the thickness [29] were subjected to blooming for 30 min and placed with the bloomed surface facing the color flex cup’s base. Triplicate readings (the cup rotates 90° in the second and third reading) for each sample were recorded for L* (lightness), a* (redness), and b* (yellowness) values, and then averaged [30]. The saturation index of chroma √ (a2 + b2) were also calculated following [31].

#### 2.1.7. Muscle pH

The right *Longissimus lumborum* (LL) muscle samples were removed from −80 °C storage and manually pulverized in liquid nitrogen using a mortar and pestle. Approximately 0.5 g of pulverized muscle tissue were collected and homogenized (Wiggen Hauser^®^ D-500, Berlin, Germany) for 30 s, in 10 mL ice-cold deionized water. Sodium iodoacetate (Merck Schuchardt OHG, Hohenbrunn, Germany) was added to inhibit further glycolysis (specifically glyceraldehyde 3-phosphate dehydrogenase) and production of lactic acid (AMSA, 2012) [29]. The resultant homogenates’ indirect pH was read using a pre-calibrated portable pH meter (Mettler-Toledo AG, Zürich, Switzerland).

#### 2.1.8. Water Holding Capacity

The water holding capacity (WHC) of the meat samples was evaluated by measuring the cooking and drip losses, following the procedures of [32]. In assessing the cooking loss, samples of LL muscles were weighed (W1), placed in polyethene bags, and vacuum packed. The samples were cooked in a preheated water bath set at 80 °C. Once the samples’ internal temperature reached 78°C, as monitored using a stabbing temperature probe (HI 145-00 thermometer, HANNA^®^ instruments, Woonsocket, RI, USA), the cooking continued for another 10 min. After removing the cooked samples from the water bath and subsequent cooling to room temperature, the samples were blotted gently dry and reweighed (W2). The percentage of cooking loss was estimated using the following formulae:

$$Cooking\;loss\;\left(\%\right)=\left(\frac{\left(Wx-Wy\right)}{Wx}\right)\times 100$$

where *Wx* is the muscle weight before water bath cooking (g) and *Wy* is the muscle weight after water bath cooking (g).

$$Drip\;loss\;\left(\%\right)=\left(\frac{\left(Wa-Wb\right)}{Wa}\right)\times 100$$

where *Wa* is the muscle weight before storage (g) and *Wb* is the muscle weight after storage (g).

#### 2.1.9. Shear Force

The meat samples used to determine cooking loss were prepared and used for evaluating the shear force values using a texture analyzer (TA. HD plus^®^, Stable Micro System, Surrey, UK) equipped with a Volodkevitch bite jaw. Calibration of the device was done at 5 kg for weight, and the speed of the blade and distance for height was set at 10 mm/s. Samples were prepared following Sazili et al. [32]. Triplicate blocks of 1 cm (height) × 1 cm (width) × 2 cm (length) were cut parallel to the direction of the muscle fibres from each sample. Each block was sheared with Volodkevitch bite jaw in the centre and perpendicular to the fibres’ longitudinal direction. Shear force values were reported as the average peak positive force of all block values of each sample. The tenderness of the meat was inversely proportional to the shear force values [33].

#### 2.1.10. Muscle Glycogen

Glycogen content in LL muscles was determined using an EnzyChrom^TM^ Glycogen Assay kit catalogue no. E2GN-100 (BioAssays Hayward, CA, USA), following the manufacturer’s instructions for the colorimetric assay. Concisely, samples were manually crushed in liquid nitrogen. Frozen crushed muscle tissue (2 g) was homogenized (Wiggen Hauser^®^ D-500, Berlin, Germany) with 10 mL ice-cold 0.1 M citrate buffer (pH 4.2) containing 0.1 M citric acid and 0.1 M sodium citrate. Then, the homogenates were centrifuged at 12,000× *g*, for 5 min, at 4 °C (Avanti^®^ J-26 XPI, BECKMAN COULTIER^®^, CA, Kraemer Blvd. Brea USA). For standard curve preparation, 5 µL of glycogen standard (50 mg/mL) was diluted with 1.245 mL deionized water to obtain 200 µg/mL glycogen standards. Thereafter, 200, 150, 100, 50, and 0 μL of 200 µg/mL glycogen standard were diluted with 0, 50, 100, 150, and 200 μL of dH_2_O to generate standards 1, 2, 3, 4, and 5, respectively. In duplicate, ten μL of 200, 150, 100, 50, and 0 µL of 200 µg/mL glycogen standard and samples were loaded into separate wells of the 96-well plate (Greiner Bio-One Frickenhausen, Germany). The volume was adjusted to 100 µL/well by adding 90 µL of working reagent (90 µL assay buffer (potassium phosphate, sodium chloride, and bovine serum albumin), 1 µL enzyme A (α-amylase and amyloglucosidase), 1 µL enzyme B (glucose oxidase), and 1 µL dye reagent (dimethyl sulfoxide)) to each standard and sample well. The plate was incubated at room temperature in the dark for 30 min before the optical density was determined at 570 nm wavelength using a Rayto RT-2100C microplate reader (Rayto Guangdong, China). The background was corrected by subtracting a 0 glycogen control from all sample readings. Then, a standard curve was generated by plotting the ∆OD_570_ against standard concentrations. The concentration of glycogen in the samples was calculated from the standard curve according to the formula:

Glycogen concentration = [(R_sample_ − R_blank_)]/Slope (µg/mL)

where R_sample_ and R_blank_ are the _OD570nm_ values of the sample and blank (Standard 5).

#### 2.1.11. Malondialdehyde Assay (MDA)

The LL muscle samples were removed from −80 °C storage and manually pulverized in liquid nitrogen using a mortar and pestle. Malondialdehyde is a secondary product of lipid peroxidation and is the major substrate in the TBARS test [34]. Measurements were taken on the 0, 1, 7 and 14 d. Lipid oxidation was measured using TBARS values according to the method of Lynch and Frei (1993) [35], modified by Mercier et al. (1998) [36]. Approximately, 1 g of meat sample was homogenized in 4 mL 0.15 M KCl + 0.1 mM BHT with Ultra-Turrax (1 min, 6000 rpm). After homogenization, 200 μL of the sample were mixed with TBARS solution, and then heated in a water bath at 95 °C for 60 min until a pink color developed. After cooling, 1 mL distilled water and 3 mL n-butyl alcohol were added to the extracts and homogenized. The mixture was centrifuged at 5000 rpm for 10 min. Absorbance of supernatant was read against an appropriate blank at 532 nm using a spectrophotometer (Secomam, Domont, France). The TBARS value was calculated from a standard curve of 1,1,3,3-tetraethoxypropane and expressed as mg malondialdehyde (MDA)/kg meat.

#### 2.1.12. Acute Phase Proteins

Alpha-1-acid glycoprotein (AGP) (Immunology Consultant’s Laboratory Inc., Portland, OR, USA, kit no. E-10AGP lot #2), and serum amyloid-A (SAA) (Tridelta Development Ltd., County Kildare, Ireland catalogue #TP802) were used to determine the appropriate enzyme-linked immunoSorbent assay (ELISA) kits.

#### 2.1.13. Statistical Analysis

The experiment was a complete randomized design (CRD). Statistical analysis was performed using the general linear models (GLM) procedure of Statistical Analysis System (SAS) package Version 9.4 software [37]. Data were analyzed with stocking density, distance, and their two-way interactions as the main effects. Where significant results were found, comparison among means was made by Duncan’s multiple range test. The statistical significance is considered at *p* < 0.05.

## 3. Results

### 3.1. Meat Color Values (L*, a*, and b* H*)

The effects of different distances and stocking density on color coordinates of LL muscle at different postmortem ageing are shown in Table 1. The L* (lightness) results showed significant interactions between distance and stocking density at one day (*p* > 0.05) and seven days (*p* > 0.05) postmortem but not at 14 days (*p* > 0.05) postmortem. At one day postmortem, distance did not affect lightness in low-SD animals. However, in medium-SD and high-SD animals, lightness was higher in animals transported for a short distance. At seven days postmortem, low-SD and medium-SD animals had significantly (*p* < 0.0001) higher lightness values than high-SD animals in long distance. Nonetheless, medium-SD animals had significantly (*p* < 0.0001) higher values in the medium-distance than low-SD and high-SD animals. Neither stocking density nor distance affected lightness (*p* > 0.05) at 14 days postmortem.

At one day postmortem, there were significant interactions between distance and stocking density for redness (a*). Medium-SD animals had significantly (*p* < 0.0001) higher a* values than low-SD and high-SD animals in long distance. Whereas, in the medium distance, the low-SD group had significantly (*p* < 0.0001) higher values than the other two groups. At seven days postmortem, long distance had significantly (*p* < 0.0001) higher a* values than the medium distance. Regardless of distance, the low-SD group had significantly (*p* < 0.0001) higher a* values than medium-SD and high-SD groups. Neither stocking density nor distance affected (*p* > 0.05) redness values at 14 days postmortem.

There were significant interactions (*p* < 0.0001) between distance and stocking density for yellowness (b*) at all ageing periods. At one day postmortem, stocking density did not affect the b* values in medium-distance transported animals. However, animals transported for long distances, high-SD and medium-SD groups had higher b* values than the low-SD group at one day postmortem. At seven and 14 days postmortems, the high-SD group had higher b* values than the medium-SD and low-SD groups in medium distances. However, medium-SD and low-SD groups had higher b* values as compared with high-SD in long distances. The ageing period had significant (*p* < 0.0001) effects on yellowness values in all groups, except those stocked at medium-SD in medium-distance transportation.

All ageing periods showed significant (*p* < 0.0001) interactions between distance and stocking distance for chroma (H*). At one day postmortem, on the one hand, high-SD and medium-SD animals had higher H* values than low-SD animals in long distances. On the other hand, low-SD had a higher H* value than high-SD and medium-SD animals in medium distances. At seven days postmortem, distance did not affect H* when animals were stocked at low SD. However, animals transported for a long-distance had high H* as compared with medium distances when animals were stocked at medium SD and high SD. At 14 days postmortem, H* of the high-SD group was higher than that of the low-SD group in medium distances. Conversely, H* of the low-SD group was higher than that of the high-SD group in those transported for long distances. The ageing period had a significant (*p* < 0.05) effect on H* in all groups of long-distance transported animals but not those transported for medium distances.

#### 3.1.1. Muscle pH

The pH of LL muscle at different postmortem ageing in Brahman cattle subjected to different stocking densities and transport distances is shown in Table 2. There were no significant interactions between distance and density (*p* > 0.05). Distance had a significant effect on the muscle pH at 0 (*p* < 0.0001), 7 (*p* = 0.0048), and 14 (*p* < 0.0001) days postmortem. Similarly, stocking density significantly affected pH values at 0 (*p* < 0.0001), 1 (*p* < 0.0001), and 7 (*p* = 0.0036) days postmortem. Long distance resulted in higher pH values than the medium distance.

#### 3.1.2. Muscle Glycogen

The effects of distance and stocking density on glycogen concentration of *Longissimus lumborum* (LL) muscle at different postmortem ageing are shown in Table 2. No significant distance × stocking density interactions were observed (*p* > 0.05). The concentration of glycogen in the muscle was significantly (*p* < 0.0001) affected by distance and stocking density. At 0, 1, 7, and 14 days postmortem, glycogen values were significantly (*p* < 0.0001) higher in animals transported for medium distances than long distances. Irrespective of the distance, the low-SD group had A substantially (*p* < 0.0001) higher glycogen concentration as compared with the medium-SD and high-SD groups.

#### 3.1.3. Water Holding Capacity

The results of the cooking loss of LL muscle at different postmortem ageing in cattle subjected to varying distances and stocking densities are illustrated in Table 3. Significant interactions (*p* < 0.0001) between the distance and density were observed at 7 and 14 days postmortems. At seven days postmortem, low-SD and medium-SD animals had higher cooking loss percentages than high-SD animals transported for medium distances. The ageing period had no significant effect (*p* > 0.05) on cooking loss in long-distance transported animals as compared with those transported for medium distances.

#### 3.1.4. Shear Force

The results for shear force values of LL muscle at different postmortem ageing in cattle subjected to varying distances and stocking density are illustrated in Table 3. Significant interactions between distance and stocking density were observed at one (*p* < 0.0001) and 14 (*p* = 0.0209) days postmortem. At one day postmortem, distance did not affect the shear force values when animals were stocked at high SD. However, long-distance transportation resulted in higher shear force values in animals subjected to stocking density at 200 kg/m^2^ and lower shear force values in animals stocked at 400 kg/m^2^ as compared with medium-distance transportation. At 14 days postmortem, medium-distance transportation resulted in higher shear force values in low-SD and high-SD animals.

Nonetheless, animals subjected to long-distance transport indicates higher shear force values in medium SD. At seven days postmortem, long-distances led to significantly (*p* < 0.0001) higher shear force values than medium distances. Regardless of distance, low-SD animals tended to have higher shear force values than high-SD animals.

#### 3.1.5. Meat Lipid Oxidation

The effects of different distance and stocking density on lipid oxidation of LL muscle at other postmortem ageing are shown in Table 4. There were significant (*p* < 0.0001) interactions between distance and stocking density on lipid oxidation of LL muscle in cattle at zero and seven days postmortem. Additionally, a significant difference was observed between ageing days in both long and medium distances. At zero days postmortem, stocking density did not affect lipid oxidation in animals transported for medium distances. However, lipid oxidation was higher in medium-SD and high-SD groups than the low-SD group transported for long distances.

#### 3.1.6. Acute Phase Proteins

The effects of distance and stocking density on alpha-1-acid glycoprotein (AGP) and serum amyloid-A (SAA tables) are shown in Table 5, Table 6, Table 7, Table 8, Table 9, Table 10 and Table 11, respectively.

The current study results indicate a preload value of 77.69 ± 0.87 ug/mL, 77.79 ± 1.23 ug/mL, and 77.95 ± 1.77 ug/mL for AGP representing low-SD, medium-SD, and high-SD groups, respectively, for both distances. Two-fold elevated AGP values after unloading were recorded, with 162.83 ± 2.98 ug/mL for low-SD, 180.93 ± 4.29 ug/mL for medium-SD, and 194.98 ± 4.73 ug/mL for high-SD groups. Additionally, the results obtained during neck cut increased by three-fold, i.e., 162.26 ± 1.20 ug/mL for low-SD, 166.82 ± 1.49 ug/mL for medium-SD, and 180.42 ± 2.42 ug/mL for high-SD groups.

The interaction between handling as affected by distance in cattle subjected to road transport is shown in Table 7. Our results showed a mean value of 78.01 ± 0.84 ug/mL for preload in animals subjected to transportation for medium-distances, 164.56 ± 5.70 ug/mL for unloading, and 164.42 ± 1.35 ug/mL at neck cut. Similarly, animals subjected to transportation for long distances had a mean value of 77.38 ± 1.56 ug/mL for preload, 194.30 ± 4.96 ug/mL during unloading, and 175.24 ± 2.01 ug/mL at neck cut.

The interaction between handling as affected by stocking densities in cattle subjected to road transport is shown in Table 8 (main effect) and Table 9. Our results showed a mean value of 76.29 ± 1.64 ng/mL for low-SD, 75.63 ± 1.51 ng/mL for medium-SD, and 75.95 ± 1.27 ng/mL for high-SD groups were recorded for preload values for SAA for both distances. Mean SAA values elevated by about two-fold after unloading with a mean of 112.78 ± 4.24 ng/mL recorded for low-SD, 140.86 ± 6.60 ng/mL for medium-SD, and 182.59 ± 11.31 ng/mL for high-SD groups, respectively. Similarly, results obtained during the neck cut showed a similar trend observed with mean values increasing by three-fold, with 142.08 ± 3.61 ng/mL for low-SD, 164.59 ± 3.50 ng/mL for medium-SD, and 192.61 ± 6.70 ng/mL for high-SD groups, respectively.

The interaction between handling as affected by distance in cattle subjected to road transport is shown in Table 10 and Table 11. Our results showed a mean value of 75.97 ± 1.32 ng/mL for preload in animals subjected to transportation for medium distances, 122.64 ± 6.50 ng/mL during unloading, and 147.11 ± 2.70 ng/mL recorded during neck cut. Similarly, animals subjected to transportation for long distances had mean values of 75.77 ± 1.08 ng/mL for preload, 168.18 ± 7.63 ng/mL during unloading, and 185.75 ± 5.15 ng/mL recorded during neck cut.

## 4. Discussion

In the present study, the effects of stocking density and distance on physicochemical properties of meat, oxidative stability, and acute-phase proteins in Brahman crossbred cattle transported by road under hot and humid tropical conditions were investigated. Distance and stocking density produced significant changes in muscle color coordinates, pH, cooking loss, shear force, glycogen, and MDA at the different postmortem ageing periods. Additionally, the results of acute-phase proteins were significantly affected by both distances and stocking densities.

Color is an essential organoleptic characteristic that influences meat acceptability and plays a significant role in visual appeal and purchase decisions [38,39]. Low acidity in the ageing period results in changes of color, structure, taste, and meat tenderness [40,41]. A decrease in lightness values is positively related to pH change and occurs at a pH of approximately 6.0 [42]. Dark muscle color or dark firm and dry meat (DFD) is a common condition encountered when animals are exposed to factors that deplete muscle glycogen levels before slaughter [43]. Additionally, DFD meat is of animal welfare consideration and of economic concern to the beef industry. There are numerous factors thought to increase the propensity of DFD in carcases, of which transportation is one [8].

In this study, there were significant interactions between distance and stocking density on color coordinates of muscle, with color values being negatively affected; darker color or redder meat often referred to as the DFD with an increase in stocking density in both medium and long-distance transport. Additionally, cattle are more susceptible to stress due to preslaughter stress and transport resulting in DFD meat. The works of Warris (2000) [8] corroborated our findings, where animals were transported for a longer duration had DFD meat. Additionally, these results agreed with those obtained by Kim et al. [17], who reported significant interactions between stocking density and transportation time on L* values in pigs. The authors found that stocking pigs at high density resulted in lower L* value, when animals were transported for a longer time than a shorter time (1 h). On the contrary, at medium and low stocking densities, animals transported for a longer time had higher L* than those freighted for a medium time.

The pH plays a primary role in determining changes in postmortem muscles’ color values [44]. A limited pH decline results in high pH (pH > 5.7) and darker meat color, while a complete and normal pH decline to 5.4–5.5 can result in a bright cherry-red meat color [45]. Thus, the differences in color coordinates of muscle, in this study, could be attributed to the higher pH values, which increased with increasing distance of transport and or high stocking densities, even though muscle pH increases with increasing stocking density. In addition, depletion of glycogen, which could be as a result of transport exhaustion, fear due to novelty, thermal extremes and other climatic factors, feed withdrawal prior to slaughter resulting in prolonged hunger, increases the susceptibility of cattle exposed to different stressors; the carcases exhibit dark firm and dry DFD condition, when slaughtered before having sufficient time to replenish their muscle glycogen reserves.

In this study, significant interactions were observed between distance and stocking density on cooking loss. Medium-distance transport and high stocking density had substantial effects on cooking loss. The current findings are similar to those reported by [18] in rabbits, where cooking loss decreased with increasing transportation duration. Ref. [46] reported increased water losses in the meat of lower ultimate pH, which could be explained by the net charge effect when the isoelectric point of muscle proteins was eventually attained [47,48]. The same authors also concluded that cooking loss was negatively associated with ultimate muscle pH. Thus, our results suggest that animals transported at high SD for a long distance have a higher cooking loss.

There were significant interaction effects for both distances on days seven and 14. No systemic trend or pattern was observed for the interactions in the shear force. Kadim et al. [49] found that meat from transported goats was tougher than that from non-transported goats. The ultimate pH of muscle significantly influences meat quality characteristics, including color, water-holding capacity, and tenderness [48,50,51]. Early postmortem meat tenderization through myofibrillar degradation has been associated with the calpain proteolytic system [52,53,54]. Calpain has the highest activity at pH 6.5, and its activity is decreased at lower pH, while pH does not have a significant effect on calpastatin inhibitory activity [55]. As a stressor, transportation causes a decline in meat pH by accelerating lactate accumulation and subsequently increases meat toughness [35].

Regarding meat quality, glycogen depletion is one of the essential metabolic changes due to preslaughter stress and the resultant inability of muscles to develop adequate acidity levels postmortem [56]. Muscle glycogen levels in live animals is an essential factor influencing the extent of postmortem glycolysis, which determines the ultimate pH. When glycogen reserves are low at the time of slaughter, less lactic acid is formed during rigour development, resulting in ultimately high pH, which negatively affects meat quality resulting in dark cuts. In this study, glycogen levels were lower in animals transported for long distances than those transported for short distances. Similar effects on muscle glycogen after a long duration of transportation have been reported in cattle [19,35] and sheep [57].

Thus, the current findings corroborate with early works conducted that suggested muscle glycogen reserves were depleted during long-distance transport. Frequent movements of muscles and energy are required to maintain animals’ balance as compared with medium-distance transport. In this study, animals transported in high SD had lower glycogen levels than those in medium SD and low SD.

Similarly, the effects of higher SD on glycogen have been reported by [58] in pigs, during transportation, mainly when the space allowance was insufficient to allow cattle and sheep to lie down during the journey. Additional energy is required to serve these demands, which impacts muscle glycogen concentration and potentially ultimately pH [59]. Thus, the lower glycogen content witnessed in cattle subjected to higher stocking density could increase energy demand.

Mechanisms controlling meat quality development are often associated with changes in the extent or rate of glycolysis, which can create an undesirable muscle pH and DFD meat. In this study, the pH values were significantly higher in animals transported for long distances than those carried for medium distances. Similarly, higher ultimate pH was also reported in cows [60,61], steers [23], rabbits [18] subjected to a long duration of transportation.

Thus, the present study results suggest that animals transported for long distances were more exhausted than those carried for medium distances, which could be explained by increased glycogenolysis, which is the biochemical breakdown of glycogen to glucose, and therefore higher pH values. The glucocorticoid content of blood is generally considered to be a useful index for the reaction of animals to environmental challenges [62]. Higher stocking density during transportation has been reported to cause more significant physiological stress than low stocking density [63].

Furthermore, muscle pH values were higher in animals freighted at high SD than those transported at medium and low SD. Our results are in contradiction with [18,23], who reported no effects of high stocking density on muscle pH in steers and rabbits, respectively. However, in steers, [23] reported increased cortisol levels with increasing stocking density. On the contrary, [64] observed no difference in cortisol levels in goats stocked at low and high densities (0.2 m^2^ vs. 0.4 m^2^/animal). A similar response was observed in Friesian steers after a four-hour journey [23].

The oxidative stability of meat is a limiting factor determining the shelf-life and safety of beef [65]. Lipid oxidation has been regarded as the leading cause of deterioration of flavor [66], the formation of rancid odors [67], discoloration [68], and the production of potentially toxic compounds in meat [69]. In oxidative conditions, the interaction of proteins with other biomolecules can lead to cross-linking and or polymerization. Thus, carbohydrates, aldehydes, and lipid oxidation of products (malondialdehyde or 4-hydroxynonenal) can react with amino groups of proteins to form fluorescent aggregates termed lipofuscin or ceroid [70].

Lipids are susceptible to oxidative damage because of the fast depletion of endogenous antioxidants after slaughter [71], during meat ageing, and storage. The dissociation of haem from myoglobin and iron from haem is a significant factor responsible for the pro-oxidative effect of proteins and lipids [62]; [72]. In lambs transported by road for short (30 min) or long (5 h) duration at different stocking densities, [13] there were no interactions between stocking density and transport time on lipid oxidation observed.

On the contrary, in the present study, there were significant interactions between distance and stocking density observed with increasing stocking density resulting in higher lipid oxidation in long distances. A possible explanation for the inconsistency between our findings and those documented by [13] could be the differences in the transportation time and the stocking density between the two studies. In the present study, the higher level of lipid oxidation observed in animals stocked at higher density and transported for a long distance is attributable to the physiological response under such stressful situations.

The results of the present study showed an increase in values of AGP and SAA with an increase in the distance, which may likely be due to several factors such as thermal extremes and longer durations on a haul, which is in agreement with the findings of [73]. Additionally, other factors contributing to this effect could be fear due to novelty, handling, and an increase in adrenal activity [22,74,75]. Furthermore, our findings agree with the works of [76], who reported an increase in APPs after exposing calves and pigs to shipping conditions and suggested that APPs could be used as biomarkers of stress and animal well-being. However, our results disagree with [77] who observed no difference with an increase in APPs measured in bulls after transportation with different stocking densities.

## 5. Conclusions

Road transport of cattle from within Malaysia sufficiently affected meat quality parameters, particularly pH, color, cooking loss, and various acute-phase proteins’ concentration. Distance, stocking density, and extreme temperature during transport can be linked with changes observed in meat. As seen by the interactions between stocking density and distance on color, cooking loss, and lipid oxidation, higher stocking affected the meat quality when animals were transported for long distances. Additionally, both AGP and SAA were affected by both distances and stocking densities. The results of the current findings highlight the impact of transportation stress on meat quality and welfare of cattle. Therefore, it is necessary to improve monitoring of cattle during transportation and while at lairage to minimize stress and to allow animals to acclimatize after a series of events. Additionally, there is a need for training of livestock handlers, truck drivers, as well as improved logistics, in order to reduce the risk of dark firm and dry (DFD) carcases and minimize animal welfare problems as a result of transportation. Finally, additional studies are required to corroborate the current findings and make valid recommendations. An important area of study could be design of truck and thermal conditions comfortable for cattle during transport.

## Figures and Tables

**Table 1:** Differences in color coordinates of *Longissimus lumborum* muscle during postmortem ageing periods in cattle subjected to different distances and stocking densities (SD) during road transport.

Color Coordinate	Ageing (Day)	Long Distance (850 km)			Medium Distance (450 km)			Level of Significance		
		High-SD (600 kg/m^2^)	Medium-SD (400 kg/m^2^)	Low-SD (200 kg/m^2^)	High-SD (600 kg/m^2^)	Medium-SD (400 kg/m^2^)	Low-SD (200 kg/m^2^)	Distance	Stocking	Distance × Stocking
No of animals		10	10	10	10	10	10			
Lightness (L)										
	1	35.20 ± 0.86 bx	31.82 ± 1.84 dz	35.79 ± 0.85 b	36.94 ± 0.59 ax	33.49 ± 0.84 cy	35.51 ± 1.82 b	0.1263	0.0003	0.0135
	7	34.83 ± 1.98 cy	37.12 ± 1.61 abx	37.60 ± 2.18 a	36.58 ± 1.24 bx	37.83 ± 1.45 ax	36.80 ± 1.09 b	0.1276	<0.0001	0.0014
	14	33.17 ± 0.23 z	33.61 ± 0.36 y	33.42 ± 0.96	34.14 ± 0.73 y	33.14 ± 0.76 y	33.69 ± 1.20	0.3150	0.1112	0.1555
	*p* value	0.0170	0.0078	0.7568	0.0008	0.0470	0.1615			
Redness (a)										
	1	15.14 ± 0.23 dx	18.88 ± 0.24 ax	14.95 ± 0.24 e	15.71 ± 0.30 cx	15.94 ± 0.27 c	17.57 ± 0.47 b	0.0002	0.0128	<0.0001
	7	13.85 ± 0.34 by	14.16 ± 0.91 by	14.96 ± 0.85 a	12.07 ± 0.70 cy	12.60 ± 0.79 c	14.26 ± 2.85 b	<0.0001	<0.0001	0.1100
	14	14.15 ± 0.33 y	15.16 ± 0.56 y	13.03 ± 0.92	14.46 ± 0.29 x	15.18 ± 0.70	14.95 ± 0.80	0.1868	0.1042	0.1090
	*p* value	<0.0001	0.0242	<0.0001	0.0182	0.1889	0.4005			
Yellowness (b)										
	1	17.86 ± 0.78 ax	16.95 ± 0.66 ay	15.43 ± 0.54 ey	16.52 ± 0.47 by	16.41 ± 0.28 b	16.31± 0.35 bcz	0.6466	0.0109	<0.0001
	7	16.01 ± 0.24 dy	19.03 ± 0.51 ax	17.70 ± 0.70 cx	19.10 ± 0.48 ax	15.92 ± 0.51 d	18.19 ± 0.71 bx	0.0003	0.0055	<0.0001
	14	15.92 ± 0.51 dy	16.50 ± 0.83 cy	16.86 ± 0.41 cby	18.23 ± 0.81 ax	6.94 ± 0.58 cb	17.05 ± 0.48 by	0.0003	0.0508	<0.0001
	*p* value	0.0005	0.0487	0.0029	0.0043	0.6393	0.0075			
Chroma (H)										
	1	28.29 ± 0.49 ax	27.49 ± 0.39 bx	21.49 ± 0.59 ey	22.82 ± 0.96 d	22.88 ± 0.64 d	26.12 ± 0.51 c	0.0056	0.0022	<0.0001
	7	21.18±0.38 cy	23.78 ± 0.54 ay	23.44 ± 0.66 ax	22.63 ± 0.32 b	20.11 ± 0.43 d	23.42 ± 0.78 a	<0.0001	<0.0001	<0.0001
	14	20.58 ± 0.91 dy	22.41 ± 0.85 by	21.34 ± 0.71 cy	23.28 ± 0.41 a	22.81 ± 0.90 ab	22.68 ± 0.55 b	0.0085	0.1238	<0.0001
	*p* value	<0.0001	0.0317	<0.0001	0.7260	0.3162	0.1619			

a–d, means in the same row with different letters are significantly different at *p* < 0.05; x–z, means in the same column with different letters are significantly different at *p* < 0.05.

**Table 2:** Differences in the glycogen content and pH values of *Longissimus lumborum* muscle during postmortem ageing periods in cattle subjected to different distances and stocking densities (SD) during road transport.

Parameters	Ageing (Day)	Long Distance (850 km)			Medium Distance (450 km)			Level of Significance		
		High-SD (600 kg/m^2^)	Medium-SD (400 kg/m^2^)	Low-SD (200 kg/m^2^)	High-SD (600 kg/m^2^)	Medium-SD (400 kg/m^2^)	Low-SD (200 kg/m^2^)	Distance	Stocking	Distance × Stocking
No of animals		10	10	10	10	10	10			
Glycogen (mg/kg meat)										
	0	1.411 ± 0.002 fw	1.464 ± 0.002 ew	1.539 ± 0.002 dw	1.655 ± 0.001 cw	1.67 ± 0.001 bw	1.753 ± 0.001 aw	<0.0001	<0.0001	0.1100
	1	0.776 ± 0.001 dx	0.799 ± 0.001 cx	0.808 ± 0.001 cx	0.826 ± 0.001 bx	0.825 ± 0.002 bx	0.837 ± 0.001 ax	<0.0001	<0.0001	0.0553
	7	0.675 ± 0.001 cy	0.687 ± 0.001 cy	0.698 ± 0.001 cy	0.702 ± 0.001 cy	0.710 ± 0.001 by	0.731 ± 0.001 ay	<0.0001	<0.0001	0.2257
	14	0.341 ± 0.001 cz	0.347 ± 0.001 cz	0.356 ± 0.002 bz	0.356 ± 0.001 bz	0.356 ± 0.001 bz	0.376 ± 0.002 az	<0.0001	<0.0001	0.1822
	*p* value	<0.0001	<0.0001	<0.0001	<0.0001	<0.0001	<0.0001			
pH (unit)										
	0	6.66 ± 0.01 ax	6.47 ± 0.02 bx	6.38 ± 0.02 bx	6.26 ± 0.02 bx	5.85 ± 0.03 cx	5.80 ± 0.04 cx	<0.0001	<0.0001	0.2849
	1	5.62 ± 0.01 az	5.54 ± 0.01 bz	5.43 ± 0.01 cz	5.59 ± 0.01 abz	5.53 ± 0.01 by	5.36 ± 0.02 cz	0.1865	<0.0001	0.5737
	7	5.75 ± 0.02 az	5.65 ± 0.01 bz	5.60 ± 0.02 bz	5.59 ± 0.01 bz	5.60 ± 0.02 by	5.52 ± 0.01 cy	0.0048	0.0336	0.3826
	14	6.12 ± 0.01 ay	6.05 ± 0.02 by	6.00 ± 0.01 by	5.87 ± 0.01 cy	5.85 ± 0.01 cx	5.76 ± 0.01 dx	<0.0001	0.0743	0.8832
	*p* value	<0.0001	<0.0001	<0.0001	<0.0001	<0.0001	<0.0001			

a–f, means in the same row with different letters are significantly different at *p* < 0.05; w–z means in the same column with different letters are significantly different at *p* < 0.05.

**Table 3:** Differences in cooking loss and shear force values of *Longissimus lumborum* muscle during postmortem ageing periods in cattle subjected to different distances and stocking densities (SD) during road transport.

Parameters	Ageing (Day)	Long Distance (850 km)			Medium Distance (450 km)			Level of Significance		
		High-SD (600 kg/m^2^)	Medium-SD (400 kg/m^2^)	Low-SD (200 kg/m^2^)	High-SD (600 kg/m^2^)	Medium-SD (400 kg/m^2^)	Low-SD (200 kg/m^2^)	Distance	Stocking	Distance × Stocking
No of animals		10	10	10	10	10	10			
Cooking loss (%)										
	1	31.84 ± 0.64 dc	30.76 ± 0.44 dc	32.28 ± 1.08 d	34.79 ± 0.67 abx	35.54 ± 0.68 a	33.28 ± 0.36 bcy	0.0003	0.2286	0.1190
	7	31.21 ± 0.21 c	30.56 ± 0.19 c	29.16 ± 0.87 d	32.44 ± 0.16 by	36.34 ± 0.33 a	36.61 ± 0.38 ax	<0.0001	0.0005	<0.0001
	14	28.27 ± 0.72 e	32.27 ± 0.46 c	32.12 ± 0.65 c	30.03 ± 0.28 dz	33.66 ± 0.43 b	34.18 ± 0.20 ay	<0.0001	<0.0001	<0.0001
	*p* value	0.0552	0.1701	0.2595	0.0005	0.0779	0.0048			
Shear force (kg)										
	1	2.36 ± 0.06 bc	1.82 ± 0.02 dy	2.91 ± 0.02 ax	2.00 ± 0.02 cz	2.18 ± 0.04 c	2.52 ± 0.03 b	0.0583	0.1800	<0.0001
	7	2.43 ± 0.09 bc	2.57 ± 0.10 bcx	2.76 ± 0.07 ax	2.29 ± 0.05 cxy	2.47 ± 0.06 bc	241 ± 0.05 bc	<0.0001	0.0030	0.1527
	14	2.18 ± 0.01 cd	2.27 ± 0.05 cx	2.14 ± 0.07 dy	2.56 ± 0.06 ax	2.15 ± 0.01 d	2.57 ± 0.03 a	0.0018	0.0143	0.0209
	*p* value	0.1620	0.0019	0.0006	0.0113	0.5555	0.8159			

a–e Means in the same row with different letters are significantly different at *p* < 0.05. x–z Means in the same column with different letters are significantly different at *p* < 0.05.

**Table 4:** Differences in the malondialdehyde content MDA (mg/kg) of *Longissimus lumborum* muscle during postmortem ageing periods in cattle subjected to different distances and stocking densities during road transport.

Ageing (Day)	Long Distance (850 km)			Medium Distance (450 km)				Level of Significance	
	High-SD (600 kg/m^2^)	Medium-SD (400 kg/m^2^)	Low-SD (200 kg/m^2^)	High-SD (600 kg/m^2^)	Medium-SD (400 kg/m^2^)	Low-SD (200 kg/m^2^)	Distance	Stocking	Distance × Stocking
No of animals	10	10	10	10	10	10			
0	0.077 ± 0.001 az	0.076 ± 0.001 az	0.053 ± 0.004 bz	0.053 ± 0.004 bz	0.051 ± 0.002 bz	0.050 ± 0.011 bz	<0.0001	<0.0001	<0.0001
1	0.114 ± 0.003 ay	0.111 ± 0.001 aby	0.109 ± 0.002 by	0.063 ± 0.008 dz	0.060 ± 0.008 cdz	0.057 ± 0.008 dz	<0.0001	0.0205	0.8322
7	0.131 ± 0.006 ay	0.121 ± 0.002 by	0.113 ± 0.002 cy	0.109 ± 0.002 dy	0.104 ± 0.002 edy	0.102 ± 0.002 ey	<0.0001	<0.0001	<0.0001
14	1.453 ± 0.007 ax	1.333 ± 0.005 bx	1.205 ± 0.009 dz	1.240 ± 0.007 cx	1.160 ± 0.007 ex	1.100 ± 0.006 ex	<0.0001	<0.0001	0.0750
*p* value	<0.0001	<0.0001	<0.0001	<0.0001	<0.0001	<0.0001			

a–e, means in the same row with different letters are significantly different at *p* < 0.05; x–z, means in the same column with different letters are significantly different at *p* < 0.05.

**Table 5:** Serum α-1 glycoprotein (AGP) as affected by handling, stocking density, and distance in road transported cattle.

Parameters	AGP
Main Effects	
Handling	
Preload	77.81 ± 0.63 c
Unload	179.44 ± 2.78 a
Slaughter	169.83 ± 1.37 b
Density	
200 kg	134.30 ± 4.87 c
400 kg	141.85 ± 5.63 b
600 kg	150.98 ± 6.42 a
Distance	
450 km	135.72 ± 4.05 b
850 km	149.01 ± 5.14 a
*p*-value	
Handling	<0.0001
Density	<0.0001
Distance	<0.0001
Handling*Density	<0.0001
Handling*Distance	<0.0001
Distance*Density	0.2100

a–c, means within columns with no common lowercase letters are significantly different at *p* ≤ 0.05. * denotes interaction.

**Table 6:** Serum α-1 glycoprotein (AGP) as affected by the interaction between handling and stocking density in road transported cattle.

Parameter	AGP		
	Handling		
Density	Preload	Unload	Slaughter
200 kg	77.69 ± 0.87 bx	162.83 ± 2.98 az	162.26 ± 1.20 ay
400 kg	77.79 ± 1.26 cx	180.92 ± 4.29 ay	166.82 ± 1.49 by
600 kg	77.95 ± 1.77 cx	194.54 ± 4.73 ax	180.42 ± 2.42 bx

a–c, means within rows subgroup with no common lowercase letters are significantly different at *p* ≤ 0.05; x–z, means within columns subgroup with no common lowercase letters are significantly different at *p* ≤ 0.05.

**Table 7:** Serum α-1 glycoprotein (AGP) as affected by the interaction between handling and distance in road transported cattle.

Parameter	AGP		
	Handling		
Distance	Preload	Unload	Slaughter
450 km	78.01 ± 0.84 bx	164.56 ± 5.70 axy	164.42 ± 1.35 ay
850 km	77.38 ± 1.56 bx	194.30 ± 4.96 ax	175.24 ± 2.01 ax

a, b, means within rows subgroups with no common lowercase letters are significantly different at *p* ≤ 0.05; x, y, means within columns subgroups with no common lowercase letters are significantly different at *p* ≤ 0.05.

**Table 8:** Serum amyloid A (SAA) as affected by handling, stocking density, and distance in road transported cattle.

Parameters	SAA
Main effects	
Handling	
Preload	75.87 ± 0.85 c
Unload	145.41 ± 3.69 b
Slaughter	166.43 ± 5.66 a
Density	
200 kg	110.38 ± 3.72 c
400 kg	126.94 ± 5.13 b
600 kg	150.38 ± 7.62 a
Distance	
450 km	115.24 ± 3.70 b
850 km	143.23 ± 5.58 a
*p*-value	
Handling	<0.0001
Density	<0.0001
Distance	<0.0001
Handling*Density	<0.0001
Handling*Distance	<0.0001
Distance*Density	<0.0001

a–c, means within columns with no common lowercase letters are significantly different at *p* ≤ 0.05. * denotes interaction.

**Table 9:** Serum amyloid A (SAA) as affected by the interaction between handling and stocking density in road transported cattle.

Parameter	SAA		
	Handling		
Density	Preload	Unload	Slaughter
200 kg	76.29 ± 1.64 cx	112.78 ± 4.24 bz	142.08 ± 3.61 az
400 kg	75.36 ± 1.51 cx	140.86 ± 6.60 by	164.59 ± 3.50 ay
600 kg	75.95 ± 1.27 cx	182.59 ± 11.31 bx	192.61 ± 6.70 ax

a–c, means within rows subgroup with no common lowercase letters are significantly different at *p* ≤ 0.05; x–z, means within columns subgroup with no common lowercase letters are significantly different at *p* ≤ 0.05.

**Table 10:** Serum amyloid A (SAA) as affected by the interaction between handling and distance in road transported cattle.

Parameter	SAA		
	Handling		
Distance	Preload	Unload	Slaughter
450 km	75.97 ± 1.32 cx	122.64 ± 6.50 by	147.11 ± 2.70 ay
850 km	75.77 ± 1.08 cx	168.17 ± 6.63 bx	185.75 ± 5.15 ax

a–c, means columns within subgroup with no common lowercase letters are significantly different at *p* ≤ 0.05; x, y, means within rows subgroup with no common lowercase letters are significantly different at *p* ≤ 0.05.

**Table 11:** Serum amyloid A (SAA) as affected by the interaction between density within distance in SAA subjected to road transport.

Parameter	SAA		
	Density (kg/sqm)		
Distance (km)	200 kg	400 kg	600 kg
450 km	99.64 ± 3.61 by	119.03 ± 6.14 ax	127.05 ± 7.99 ay
850 km	121.13 ± 3.61 bx	134.85 ± 5.13 bx	173.72 ± 11.87 ax

a, b, means within rows subgroup with no common lowercase letters are significantly different at *p* ≤ 0.05; x, y, means within columns subgroup with no common lowercase letters are significantly different at *p* ≤ 0.05.

## Data Availability

The data presented in this study are available on request from the corresponding author. The data are not publicly available due to ongoing research.
